# Supplementary material for: Lysimachia mauritiana Lam. Extract Alleviates Airway Inflammation Induced by Particulate Matter Plus Diesel Exhaust Particles in Mice
Source: Nutrients. 2024 Oct 31;16(21):3732. doi: 10.3390/nu16213732 (PMC11547324; doi:10.3390/nu16213732)

Original western blot of lung tissue – triplicate

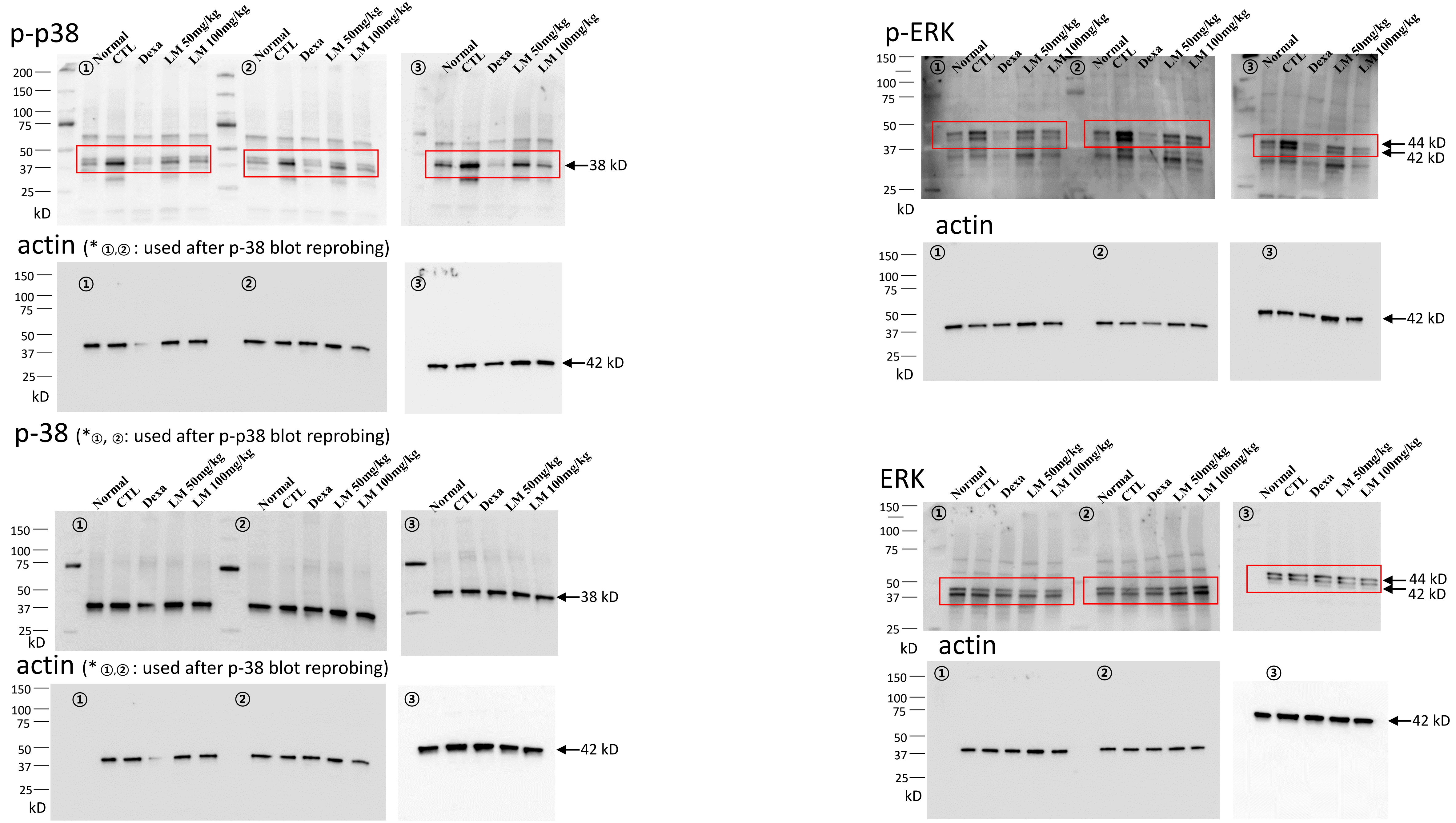

Original western blot of lung tissue – triplicate

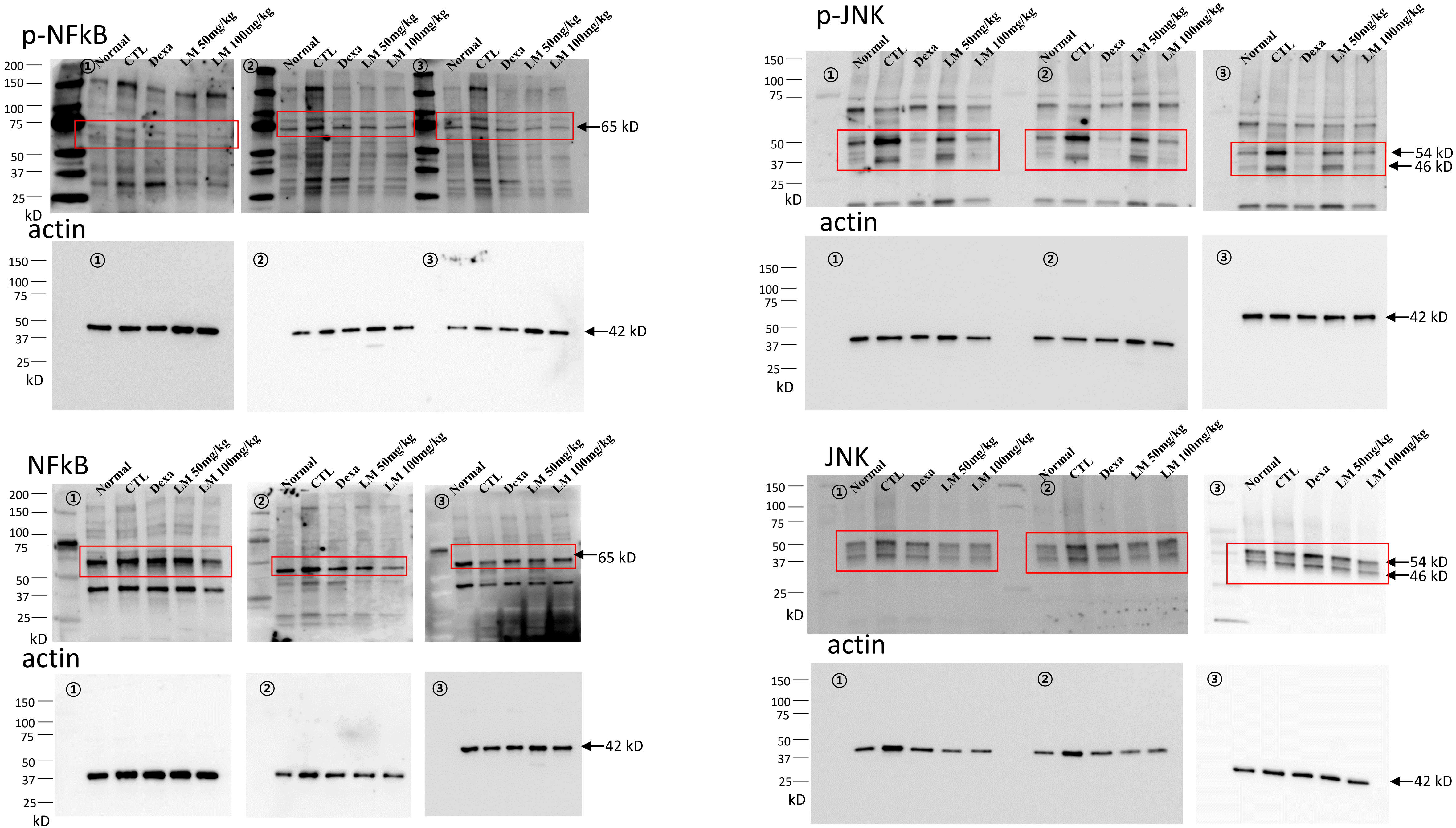

Supplement: Supplementary file 1 [file nutrients-16-03732-s001.zip › nutrients-3245509-supplementary.pdf]
